# Diverticule de Meckel compliqué d'un abcès rétro péritonéal: à propos d'un cas rare

**DOI:** 10.11604/pamj.2014.19.195.4379

**Published:** 2014-10-23

**Authors:** Hicham El Bouhaddouti, Khalid El Haoudi

**Affiliations:** 1 Service de Chirurgie Viscérale, CHU Hassan II, Fès, Maroc

**Keywords:** Diverticule de Meckel, abcès rétro péritonéal, anomalie congénitale, Meckel diverticulum, retroperitoneal abscess, birth defect

## Image en medicine

Le diverticule de Meckel est une anomalie congénitale résultant d'une résorption incomplète du canal omphalo-mésentérique, pouvant se compliquer dans 4% des cas. Il s'agit d'une patiente âgée de 48 ans, sans antécédent pathologique, admise aux urgences pour des douleurs abdominales généralisées à point de départ hypogastrique. L'examen clinique trouvait une sensibilité abdominale généralisée chez une patiente fébrile à 38,7 c. Le bilan biologique avait montré une hyperleucocytose à 18000/mm^3^, avec une CRP à 130 mg/l. L'échographie avait objectivé un épanchement intra péritonéal de moyenne abondance. Un complément scannographique avait mis en évidence une énorme collection rétro péritonéale refoulant le colon gauche et l'intestin grêle, avec une distension grêlique (A). La patiente a été opérée par laparotomie médiane, avec découverte d'un volumineux abcès rétro péritonéal en rapport avec un diverticule de Meckel situé à 90 cm de la valvule iléo caecal, et perforé au niveau de sa pointe dans le rétro péritoine (B). On a réalisé une résection grêlique emportant le diverticule avec double stomie grêlo- grêlique à la Bouilly-Walkman associée à une aspiration de l'abcès puis lavage et drainage péritonéal large (C). Les suites post opératoires ont été simples, avec rétablissement de la continuité digestive après deux mois. L'étude anatomo pathologique de la pièce de résection est revenue en faveur d'une diverticulite Chronique sans signe de malignité.

**Figure 1:**
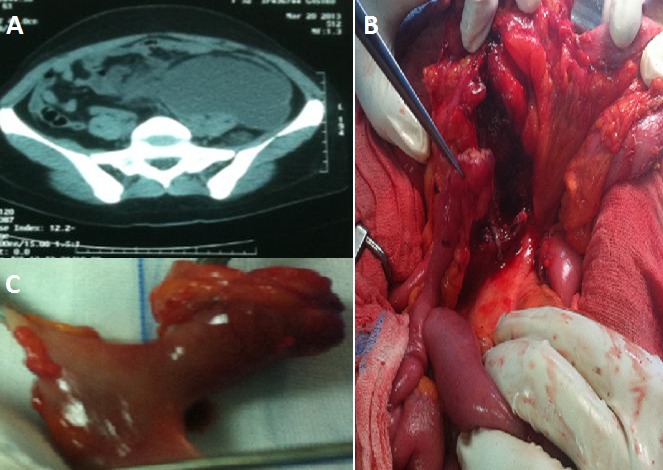
A) aspect scannographique de l'abcès rétro péritonéal; B) aspect per opératoire du diverticule de Meckel perforé en rétro péritonéal; C) pièce de résection grêlique emportant le diverticule de Meckel

